# Epidemiology, clinical relevance and prognosis of staphylococci in hospital-acquired postoperative intra-abdominal infections: an observational study in intensive care unit

**DOI:** 10.1038/s41598-021-85443-8

**Published:** 2021-03-15

**Authors:** Kévin Boussion, Nathalie Zappella, Nathalie Grall, Lara Ribeiro-Parenti, Grégory Papin, Philippe Montravers

**Affiliations:** 1 Hôpitaux de Paris, Department of Anesthesiology and Critical Care Medicine, DMU PARABOL Bichat-Claude Bernard Hospital, 46 rue Henri Huchard, 75018 Paris, France; 2 Hôpitaux de Paris, Department of Bacteriology, Bichat-Claude Bernard Hospital, Paris, France; 3 Hôpitaux de Paris, Department of General, Esogastric and Bariatic Surgery, Bichat-Claude Bernard Hospital, Paris, France; 4 Université de Paris, Paris, France; 5 INSERM UMR 1137, IAME, Paris, France; 6 Inserm UMR1149, Paris, France; 7 INSERM UMR1152, ANR-10-LABX-17, Paris, France

**Keywords:** Clinical microbiology, Infectious-disease diagnostics, Gastrointestinal diseases, Infectious diseases, Bacterial infection

## Abstract

The pathogenic role of staphylococci in hospital-acquired postoperative intra-abdominal infections (HAIs) has never been evaluated. In a tertiary care university hospital, we assessed the clinical characteristics and outcomes of patients admitted to the intensive care unit for HAIs according to the presence of staphylococci (S-HAI) or their absence (nS-HAI) in peritoneal cultures. Patients with S-HAIs were compared to nS-HAIs patients. Overall, 380 patients were analyzed, including 87 (23%) S-HAI patients [29 *Staphylococcus aureus* (Sa-HAIs) and 58 coagulase-negative staphylococci (CoNS-HAIs)]. The clinical characteristics did not differ between the S-HAI and nS-HAI patients. Adequacy of empirical anti-infective therapy was achieved less frequently in the staphylococci group (54 vs 72%, respectively, p < 0.01). The 90-day (primary endpoint) and one-year mortality rates did not differ between these groups. The S-HAI patients had decreased rates of postoperative complication (p < 0.05). The adjusted analysis of the clinical outcomes reported a decreased frequency of surgical complications in the staphylococci group (OR 0.43, 95% CI [0.20–0.93], p = 0.03). While the trends toward decreased morbidity criteria were observed in S-HAI patients, the clinical outcomes were not different between the CoNS-HAI and Sa-HAI patients. In summary, our data are not substantial enough to conclude that staphylococci exhibit no pathogenicity in HAIs.

## Introduction

Staphylococci are identified as frequent nosocomial pathogens in intensive care units (ICU)^1,2^. These microorganisms are responsible for invasive infections, including bacteremia, pneumonia, and postoperative infections. The presence of *Staphylococcus aureus* is associated with local complications such as abscesses or tissue destruction and poor prognosis. Coagulase-negative staphylococci (CoNS), usually considered as non-pathogenic, are also regularly described in documented infections^3^.

Intra-abdominal infections (IAIs) are one of the main causes of postoperative infections leading to ICU admission^1,2^ and are associated with high mortality rates^4,5^*.* Although a large number of microorganisms are hosted in the digestive tract, only a few are found in peritoneal fluid cultures and have proven pathogenicity, such as *Enterobacterales* and anaerobes^4^. Studies investigating the role of staphylococci in IAIs are limited to patients treated with peritoneal dialysis and complicated by staphylococcal catheter infections^6,7^. While the role of *S. aureus* in secondary peritonitis has been poorly studied^8^, that of CoNS species have ever been evaluated. The only published data have shown staphylococci-positive peritoneal cultures ranging from 7% up to 22% of patients^9–11^. In the last large study published by Blot et al*.,* staphylococci were cultured from 10% of the 1982 patients^5^. The prevalence of staphylococci was similar in both community- and hospital-acquired IAIs.

As a result, the pathogenicity of staphylococci remains debated in IAIs, as well as the need for antibiotic therapy, especially for CoNS. The objective of this study was to compare the epidemiological, clinical, microbiological and prognostic characteristics of patients admitted to the ICU for hospital-acquired postoperative intra-abdominal infections (HAIs) according to the presence or absence of staphylococci in their peritoneal cultures. In addition, the respective portions of *S. aureus* and CoNS were evaluated in terms of clinical presentation, therapeutic consequences and outcome.

## Methods

### Study population

This observational, monocentric, cohort study, was performed in a tertiary care university 800 beds hospital. All consecutive patients admitted to our surgical ICU between January 1999 and October 2017 for a first episode of HAI were included in a prospective database. Patients were included if they underwent a surgical intervention for HAI with positive cultures of peritoneal surgical samples yielding at least one microorganism. This procedure could be decided as part of the management during their ICU stay or could be the reason for ICU admission. Then, the medical records were retrospectively reviewed. Patients presenting with a superinfection of necrotizing pancreatitis, mesenteric ischemia without bowel perforation or HAI without positive microbiological cultures were excluded.

All methods were performed in accordance with the relevant guidelines and regulations of French law. The protocol was designed in accordance with the local institutional review boards which waived the need for signed informed consent due to the retrospective nature of our study (Comité d’Evaluation de l’Ethique des projets de Recherche Biomédicale, CEERB CHU Bichat, Paris Diderot University, Paris, France, agreement n°10-008). The collected data were declared to the Commission Nationale de l’Informatique et des Libertés (CNIL, declaration number 1413211v1).

### Data collection

Demographic data, such as age, sex, comorbidities (malignancy, diabetes mellitus, immunosuppression defined as ongoing chemotherapy, and prolonged steroid or immunosuppressive therapy) were recorded. The severity of comorbidities was stratified according to the McCabe score into non-fatal and fatal underlying diseases (sum of ultimately and rapidly fatal diseases)^12^. The characteristics of the initial surgery (emergency and wound classification^13^) and the presence of antimicrobial therapy prior to HAI surgery were collected.

At the time of reoperation for HAI, the source of the infection and its anatomical location were noted. The SOFA (Sequential Organ Failure Assessment)^14^ and SAPS II (Simplified Acute Physiology Score)^15^ scores were obtained at the time of reoperation. Organ failure was defined by a score > 2 for the corresponding SOFA category.

### Microbiological examination

Peritoneal samples were systematically collected during surgery and sent to the microbiology laboratory. Samples were processed according to the laboratory standard methods. Plates were incubated for 48 h at 35 °C. All morphologically distinct colonies were identified by standard bacteriologic techniques and tested for antibiotic susceptibility by the disk diffusion method according to the European Committee on Antimicrobial Susceptibility Testing (EUCAST)^16^. Blood cultures were systematically collected within the 24 h prior to and after surgery. All blood culture bottles were handled using the laboratory’s blood culture system (BD BACTEC; Becton–Dickinson). Multidrug-resistant (MDR) bacteria were defined as those resistant to three or more antimicrobial classes^17^.

### Anti-infective therapy

Empirical anti-infective therapy (EAT) was started at the time of reoperation for HAI. The choice of therapy considered the severity of the case, previous antibiotic therapies, and local epidemiology. According to French guidelines, EAT included a combination of broad-spectrum beta-lactams, such as piperacillin/tazobactam or imipenem/cilastatin (depending on initial severity) combined with aminoglycosides ± vancomycin^18^. Empirical antifungal agents were administered when a high risk of fungal infection was suspected and then adapted to the results of peritoneal cultures^18^. EAT was considered adequate if all the microorganisms isolated from the peritoneal samples were susceptible to at least one of the antibiotics used.

Documented anti-infective therapy based on antibiotic susceptibility of the cultured microorganisms was recorded, including escalation, de-escalation, and total duration of therapy^19–22^.

### Clinical outcomes

The primary outcome was the risk of 90-day mortality following the diagnosis of HAI after ajustment on confounding factors. Secondary outcome were persistent sepsis^23,24^, additional reoperations for the persistence of the initial infection or superinfection and their delay (first reoperation), surgical (hemorrhage, infection, digestive complications related or not to the initial surgery) and medical (cardiovascular, infectious, neurologic, renal, respiratory, thromboembolic complications) complications during the ICU stay, duration of mechanical ventilation and ICU stay, ICU and hospital mortality rates, and one-year mortality after the diagnosis of HAI.

### Statistical analysis

The demographic characteristics and therapeutic consequences of the patients were compared according to the peritoneal cultures: staphylococci-positive (S-HAIs) or without staphylococci (nS-HAIs). A subgroup analysis was performed according to the presence of *S. aureus* (Sa-HAIs) or coagulase-negative staphylococci (CoNS-HAIs). Patients with both Sa-HAIs and CoNS-HAIs cultures were considered to have Sa-HAIs. The results were expressed as medians and [25^th^–75th percentiles] for the quantitative variables and as absolute values and proportions (%) for qualitative values. Quantitative values were compared by Wilcoxon test or Student’s t-test. Qualitative data were compared by the chi-square test or Fisher’s exact test. Missing data were systematically mentioned.

Survival outcomes were estimated using Kaplan–Meier analysis and compared by logrank test and by a multivariate Cox model. Results were expressed with adjusted hazard ratio (aHR). Binary outcomes were compared by chi-square test and multivariate logistic regression. Results were expressed with adjusted odd ratio (aOR). Adjustment factors were sex, age, SOFA score at the time of reoperation, adequacy of EAT and the year of inclusion after performing the imputation with median. The results are reported with 95% confidence intervals (CI). Statistical analysis was performed with SAS 9.4 (SAS Institute, Cary, NC, USA). A p value < 0.05 was considered significant.

## Results

### Study population

Overall, 380 patients were admitted to our ICU for the diagnosis of HAI, including 87 (23%) patients with positive staphylococci cultures of the peritoneal fluid. Among these 87 patients, 29 (33%) had *S. aureus* positive peritoneal culture (Sa-HAIs) and 58 (67%) patients with CoNS-positive peritoneal cultures (CoNS-PIs). Two patients with both *S. aureus* and CoNS-positive peritoneal cultures were considered to have Sa-HAIs.

The demographic characteristics were not different between S-HAIs and nS-HAIs (Table 1). No change was observed in the case-mix during the study period (see Additional File 1 for Supplementary Results). At the time of reoperation for HAI, a gastroduodenal source of infection was more frequent in patients with staphylococci, while these patients had a lower SOFA score than nS-HAI patients (Table 1). The temporal distributions of inclusions were not different between the nS-HAI and S-HAI groups (years of inclusion of the median and [25th–75th percentiles] patients: 2004 [2001–2011] and 2005 [2002–2008], respectively, p > 0.05). The 90-day mortality rates were not different according to the period of admission (p > 0.05).

**Table 1:** Clinical characteristics of the study population.

Variables	nS-HAI n = 293		S-HAI n = 87	CoNS-HAI n = 58	Sa-HAI n = 29	Missing data
**Demographic characteristics and comorbidities**						
Age (years)	61 [50–72]		62 [45–77]	63 [46–77]	57 [37–77]	–
Male gender	164 (56)		42 (48)	32 (55)	10 (34)	–
Fatal underlying diseases	91 (31)		31 (36)	25 (43)	6 (21)^†^	–
Immunosuppression	101 (34)		27 (31)	19 (33)	8 (28)	–
Cancer	111 (38)		27 (31)	20 (34)	7 (24)	–
Diabetes mellitus	58 (20)		11 (13)	4 (7)	7 (24)^†^	–
**Initial surgery**						
Emergency procedure	106 (36)		39 (45)	30 (52)	9 (32)^†^	–
Septic or contaminated surgery	115 (39)		33 (38)	25 (43)	8 (28)	–
Antibiotics before reoperation for HAI	181 (62)		53 (61)	34 (62)	11 (38)^†^	2/0/0/0
Interval between initial surgery and reoperation (days)	7 [4–12]		7.5 [4–10]	7 [5–11]	8 [3–10]	2/1/1/0
**Severity criteria at the time of reoperation**						
SAPS II score	49 [37–60]		46 [33–57]	47 [32–58]	44 [33–56]	–
SOFA score	8 [5–10]	7 [4–9]*		7 [4–9]	7 [4–10]	–
Hemodynamic failure	197 (67)		53 (61)	36 (62)	17 (59)	–
Respiratory failure	141 (48)		32 (37)	23 (40)	9 (31)	–
Kidney failure	83 (28)		21 (24)	12 (21)	9 (31)	–
**Source of postoperative peritonitis and surgical observations**						
Anastomotic leak	107 (37)		26 (30)	16 (28)	10 (34)	–
Bowel perforation	92 (31)		31 (36)	19 (33)	12 (41)	–
Abscess	47 (16)		18 (21)	14 (24)	4 (14)	–
Gastroduodenal source	52 (18)		26 (30)*	16 (28)	10 (34)	7/1/1/0
Small bowel source	75 (26)		18 (21)	12 (21)	6 (21)	7/1/1/0
Colonic or rectal source	79 (28)		21 (24)	12 (21)	9 (31)	7/1/1/0
Generalized peritonitis	76 (26)		18 (21)	13 (22)	5 (17)	7/1/1/0

*HAI* Hospital-acquired postoperative intra-abdominal infection, *nS-HAI* infection without peritoneal staphylococci culture, *S-HAI* infection with positive staphylococci peritoneal cultures, *Sa-HAI* infection with *Staphylococcus aureus* positive peritoneal culture, *CoNS-HAI* infection with coagulase-negative staphylococcus positive peritoneal culture, *SAPS II* Simplified Acute Physiology Score II, *SOFA* Sequential Organ Failure Assessment.

[25th–75th]: [25th–75th percentiles].

*p < 0.05 between the nS-HAI and S-HAI groups; ^†^p < 0.05 between the CoNS-HAI and Sa-HAI groups.

The microbiological results are presented in Table 2. In the staphylococci group, only 5 (6%) patients had monomicrobial peritoneal samples, CoNS in all 5 cases. In addition, 55 methicillin-resistant staphylococci were cultured, including 9 *S. aureus* and 46 CoNS. Decreased proportions of enterococci in peritoneal cultures were observed in the staphylococci group (36%, n = 31 vs 53%, n = 154, p < 0.01). MDR bacteria were more frequently found in the S-HAI and CoNS-HAI groups (Table 2). If methicillin-resistant staphylococci were no longer included in the MDR bacteria count, the proportions of peritoneal samples including at least one MDR bacterium were thus not different between nS-HAIs (n = 92, 33%) and S-HAIs (n = 33, 38%) nor between Sa-HAIs (n = 9, 31%) and CoNS-HAIs (n = 24, 41%). The proportions of Gram-positive MDR bacteria isolated in peritoneal cultures were also not different between nS-HAIs (n = 33, 11%) and S-HAIs (n = 7, 8%) nor between Sa-HAIs (n = 1, 3%) and CoNS-HAIs (n = 6, 10%). Among the 13 bacteremic patients with S-HAIs, 6 of them had the same staphylococcus strain cultured from the peritoneal sample and 7 involved other microorganisms. No difference was observed between the different groups regarding the bacteremia results, except that CoNS bacteremia was found more frequently in the staphylococci group than in the nS-HAI group.

**Table 2:** Microbiological results of the four groups of patients with HAIs.

Variables	nS-HAI n = 293	S-HAI n = 87	CoNS-HAI n = 58	Sa-HAI n = 29
**Blood sample**				
Bacteremia	60 (20)	13 (15)	9 (16)	4 (14)
Gram-negative bacilli	30 (10)	5 (6)	3 (5)	2 (7)
* Enterobacterales*	24 (8)	4 (5)	2 (3)	2 (7)
Nonfermenting Gram-negative bacilli	5 (2)	1 (1)	1 (2)	–
Gram-positive cocci	15 (5)	7 (8)	6 (10)	1 (3)
* Enterococcus *spp.	3 (1)	–	–	–
* Staphylococcus aureus*	4 (1)	1 (1)	–	1 (3)
Coagulase-negative staphylococcus	1 (0.3%)	5 (6)**	5 (9)	–
* Streptococcus *spp.	7 (3)	2 (2)	2 (3)	–
Anaerobic	11 (4)	3 (3)	2 (3)	1 (3)
Fungi	6 (2)	–	–	–
**Peritoneal sample**				
Polymicrobial	234 (80)	82 (94)**	53 (91)	29 (100)
Gram-negative bacilli	218 (74)	66 (76)	41 (70)	25 (86)
* Enterobacterales*	206 (70)	58 (67)	33 (57)	25 (86) ^††^
* Escherichia coli*	139 (47)	31 (36)	16 (28)	15 (52) ^†^
* Klebsiella *spp.	38 (13)	10 (11)	4 (7)	6 (21)
* Enterobacter *spp.	43 (15)	19 (22)	13 (22)	6 (21)
Nonfermenting Gram-negative bacilli	45 (15)	16 (19)	11 (19)	5 (17)
* Pseudomonas aeruginosa*	42 (14)	12 (14)	8 (14)	4 (14)
Gram-positive cocci	198 (68)	87 (100)**	58 (100)	29 (100)
* Enterococcus *spp.	154 (53)	31 (36)**	24 (41)	7 (24)
* Enterococcus faecium*	58 (20)	7 (8)*	18 (31)	6 (21)
* Enterococcus faecalis*	89 (30)	24 (28)	6 (10)	1 (3)
* Streptococcus *spp.	62 (21)	22 (25)	8 (14)	14 (48) ^††^
Anaerobic	68 (23)	23 (26)	12 (21)	11 (38)
Fungi	108 (37)	33 (38)	22 (38)	11 (38)
* Candida albicans*	75 (26)	22 (25)	15 (26)	7 (24)
≥ 1 MDR bacteria	92 (31)	61 (70)**	48 (83)	13 (45) ^††^
Gram-negative MDR bacteria	69 (24)	28 (32)	21 (36)	7 (24)
Gram-positive MDR bacteria	33 (11)	56 (64)**	47 (81)	9 (31) ^††^

*nS-HAI* infection without staphylococci peritoneal culture, *S-HAI* infection with staphylococci positive peritoneal cultures, *Sa-HAI* infection with *Staphylococcus aureus* positive peritoneal culture, *CoNS-HAI* infection with a coagulase-negative staphylococcus positive peritoneal culture, *MDR* multidrug-resistant.

No missing data.

*p < 0.05 and **p < 0.01 between the nS-HAI and S-HAI groups; ^†^p < 0.05 and ^††^p < 0.01 between the CoNS-HAI and SA-HAI groups.

Anti-infective therapy features are displayed in Table 3. Inadequacy of EAT was frequently observed in the S-HAI group (n = 40, 46%), which was explained in 26 cases by staphylococci not being targeted (defect solely on staphylococci [n = 14] or involving staphylococci and at least another organism [n = 12]). During the documented therapy, 38/43 (88%) of the patients with staphylococci received vancomycin to target methicillin-resistant staphylococci. In addition, 31/87 (36%) patients in the staphylococci group required antibiotic escalation for documented therapy vs 48/293 (16%) in the nS-HAI group (p < 0.01).

**Table 3:** Anti-infective therapies of the four groups of patients with HAI.

Variables	nS-HAI n = 293	S-HAI n = 87	CoNS-HAI n = 58	Sa-HAI n = 29	Missing data
**Empirical anti-infective therapy**					
Monotherapy	57 (19)	16 (18)	12 (21)	4 (14)	–
Vancomycin	129 (44)	40 (46)	25 (43)	15 (52)	–
Adequate EAT	211 (72)	47 (54)**	28 (48)	19 (66)	–
**Documented anti-infective therapy**					
Monotherapy	109 (37)	24 (28)	17 (29)	7 (24)	–
Carbapenem	61 (21)	24 (28)	20 (34)	4 (14) ^†^	–
Piperacillin/tazobactam	98 (33)	24 (28)	12 (21)	12 (41) ^†^	–
Vancomycin	68 (23)	43 (49)**	31 (53)	12 (41)	–
Aminoglycosides	39 (13)	14 (16)	8 (14)	6 (21)	–
Duration (days)	10 [7–14]	10 [10–14]	10 [8–14]	10 [10–15]	1/0/0/0
De-escalation	175 (60)	43 (49)	23 (40)	20 (69)^††^	–
Escalation	48 (16)	31 (36)**	26 (45)	5 (17)^†^	–

*nS-HAI* infection without staphylococci peritoneal cultures, *S-HAI* infection with positive staphylococci peritoneal culture, *Sa-HAI* infection with *Staphylococcus aureus* positive peritoneal culture, *CoNS-HAI* infection with coagulase-negative staphylococci peritoneal culture, *EAT* empirical anti-infective therapy, *HAI* hospital-acquired postoperative intra-abdominal infection, *ICU* intensive care unit.

[25th–75th]: [25th–75th percentiles].

°Results obtained in survivors.

**p < 0.01 between the nS-HAI and S-HAI groups; ^†^p < 0.05 and ^††^p < 0.01 between the CoNS-HAI and Sa-HAI groups.

### Clinical outcomes

**Figure 1:**
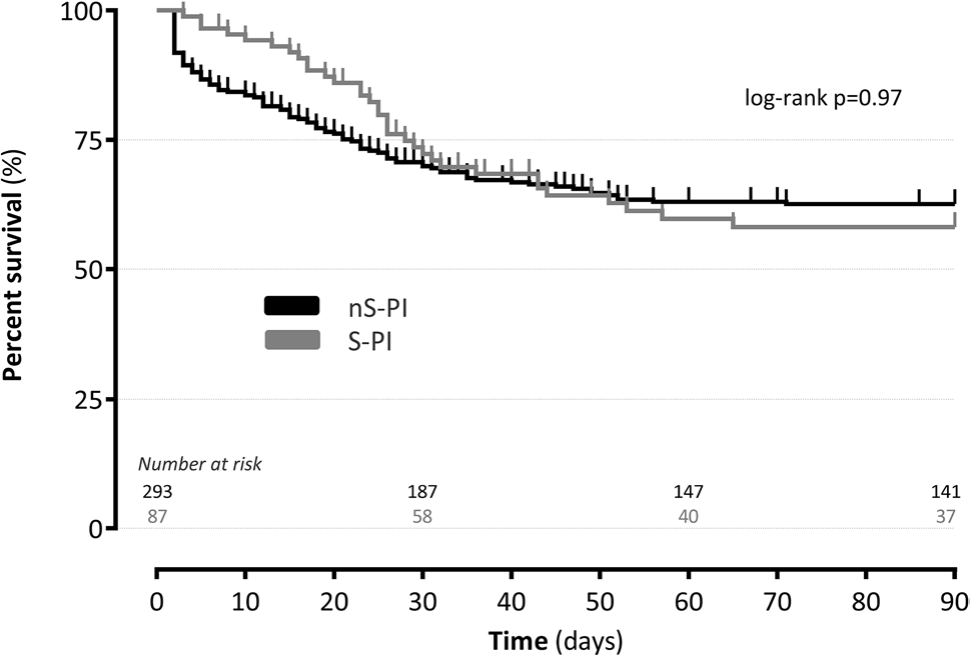
Kaplan–Meier survival estimates of the S-HAI and nS-HAI groups during the first 90 postoperative days. *nS-HAI* infection without peritoneal staphylococci cultures, *S-HAI* infection with staphylococci peritoneal cultures.

The estimated 90-day mortality were not different between nS-HAI and S-HAI groups (p > 0.05), even after adjustment (aHR = 1.56 [95% CI 0.86–2.83], p > 0.05) (Fig. 1). At one year, mortality estimated were 42% [95% CI 37–49] for nS-HAI group and 51% [95% CI 40–63] for S-HAI group (p > 0.05). When compared to the nS-HAI patients, the patients with staphylococci-positive peritoneal cultures had decreased frequencies of persistent sepsis (p < 0.05), reoperation (p < 0.05), and surgical complications (p < 0.05) (Table 3). Interval between surgery for HAI and reoperation was significantly shorter for nS-HAI groups (4 [2–8] versus 6 [4–9] days, p < 0.05). Duration of mechanical ventilation and ICU stay were similar. The adjusted analysis of secondary outcomes reported a decreased frequency of surgical complications in the staphylococci group (Table 4).

**Table 4:** Secondary outcomes of the four groups of patients with HAI.

Selected criteria	nS-HAI n = 293	S-HAI n = 87	Adjusted analysis		CoNS-HAI n = 58	Sa-HAI n = 29	Adjusted analysis	
			aOR [95% CI]	p			aOR [95% CI]	p
Persistent sepsis	164 (56)	38 (44)*	0.72 [0.42–1.23]	NS	27 (47)	11 (38)	1.20 [0.43–3.39]	NS
Reoperation	135 (46)	35 (40)	0.90 [0.52–1.51]	NS	23 (40)	12 (41)	0.27 [0.29–2.13]	NS
Surgical complications	64 (22)	9 (10)*	0.43 [0.20–0.93]	0.03	5 (9)	4 (14)	0.51 [0.12–2.18]	NS
Medical complications	45 (15)	14 (16)	1.06 [0.55–2.03]	NS	9 (16)	5 (17)	0.61 [0.14–2.68]	NS
ICU mortality	95 (32)	24 (28)	1.05 [0.56–1.95]	NS	18 (33)	6 (21)	0.14 [0.45–4.65]	NS
Hospital mortality	99 (34)	26 (30)	1.11 [0.60–2.05]	NS	19 (34)	7 (24)	1.23 [0.38–3.90]	NS

*HAI* Hospital-acquired postoperative intra-abdominal infection, *nS-HAI* infection without staphylococci peritoneal cultures, *S-HAI* infection with staphylococci peritoneal cultures, *ICU* intensive care unit, *OR* odds ratio, *aOR* odds ratio adjusted to age, sex, and SOFA score at the time of reoperation; adequacy of empirical anti-infective therapy; and the year of inclusion, *CI* confidence interval, *NS* non-significant;

*p < 0.05 between the nS-HAI and S-HAI groups.

### Subgroups analysis of peritoneal cultures with *S. aureus* and coagulase-negative staphylococci

The clinical characteristics of the patients with Sa-HAI and CoNS-HAI are presented in Table 1. Despite differences in terms of comorbidities, diabetes mellitus and previous antibiotic selection pressure, the severity scores at the time of reoperation were not different. Most cases were associated with polymicrobial infections (Table 2). The adequacy of EAT was not different between the groups. The frequency of de-escalation was predominantly performed in patients with *S. aureus* isolates (Table 3). Among the 87 staphylococci-positive patients, 16 (28%) among the 58 patients that had CoNS samples were not targeted by the documented antibiotic therapy, including 15 methicillin-resistant strains, while all the patients with *S. aureus* strains were efficiently treated.

The estimated 90-day mortality were not different Sa-HAI and CoNS-HAI subgroups 29% [95% CI 16–50] *versus* 49% [95% CI 36–64] (p > 0.05), even after adjustment (aHR = 0.50 [95% CI 0.73–3.70], p > 0.05). The secondary outcomes are reported in Table 4. In the subgroup of CoNS-HAIs, treated or untreated peritoneal CoNS were not associated with the 90-day mortality (p > 0.05). A comparison was made in removing the two patients with both *S. aureus* and CoNS-positive peritoneal cultures from the study population without showing any difference in the trends reported here (see Additional File 1 for Supplementary Results).

## Discussion

This study is one of the few to focus specifically on ICU patients admitted for HAIs with positive staphylococci peritoneal cultures. Only minor differences in clinical presentation were observed on admission compared to other patients. Adequacy of anti-infective therapy was less frequently achieved in patients with staphylococci-positive cultures. Unexpectedly, the S-HAI group had decreased morbidity (less persistent sepsis, fewer reoperation rate, and fewer surgical complications). However, no difference in hospital stays or survival was observed either between S-HAI and nS-HAI or between Sa-HAI and CoNS-HAI.

In our cohort, the prevalence of staphylococci in postoperative intra-abdominal infections was high compared to that observed in other reports, in which 9 to 12% of staphylococci-positive peritoneal samples were usually reported^5,23,24^. The demographic and clinical characteristics and initial severity were similar in patients with and without positive staphylococci peritoneal cultures, except that diabetes was more frequently observed in the Sa-HAI group, which is a well-established risk factor for *S. aureus* infections^25^. However, this link has been rarely reported in patients with intra-abdominal infections.

In our S-HAI cohort, lower proportions of enterococci, especially *Enterococcus faecium*, were reported. This observation suggests some kind of competition between Gram-positive cocci, which is an issue that has not been previously reported. Interestingly, no obvious link was reported between staphylococci and candida, while interactions with *Candida albicans* are well known for their ability to form persistent biofilms in the host or on medical devices and can lead to increased morbidity and mortality^26^. However, this is consistent with the fact that the development of biofilm in intraperitoneal infections has never been proven, except for peritonitis due to dialysis catheter infection. This comment is also valid for staphylococci and nonfermenting Gram-negative microorganisms, since collaborations between *S. aureus* and *Pseudomonas aeruginosa* have also been reported^27^. Lower proportions of *Enterobacterales*, *Escherichia coli* and *Streptococcus *spp. in the CoNS-HAIs compared to the Sa-HAIs may be explained by the fact that there was more ongoing antibiotics at the time of reoperation for PIs in this group.

Adequacy of EAT was decreased in the staphylococci group, mainly because of high proportions of methicillin-resistant staphylococci. These methicillin-resistant microorganisms required more combination therapy and more frequently included vancomycin as the documented antibiotic therapy. This difference was not found in the CoNS-HAIs vs Sa-HAIs comparison. Indeed, even if there were more methicillin-resistant staphylococci in the CoNS group, more than a quarter of them were not targeted by the definitive antibiotic therapy. There was more escalation of antibiotic therapy in the S-HAI and CoNS-HAI groups and less de-escalation in the CoNS-HAI group. The issue of de-escalation and escalation of antibiotics is debatable in the absence of standard definitions, resulting in variable interpretations between prescribers^28^. However, our criteria included both the spectrum of molecules used and their number. This approach makes it possible to consider the effects on both Gram-negative and Gram-positive bacteria.

The ICU mortality rate of patients with positive staphylococci cultures was similar to that observed with other microorganisms. Interestingly, morbidity criteria, such as persistent sepsis and surgical complications, seem to be less frequently reported in staphylococci patients contrary to what was formerly reported in a study focusing on methicillin-resistant *S. aureus* during HAIs^8^. A possible explanation for these results could be the competition within the peritoneal space between staphylococci and enterococci, and these organisms are thought to induce increased rates of morbidity and complications^29–31^. Thus, we can hypothesize that decreased morbidity rates could be linked to lower proportions of enterococci in the S-HAI group.

Unexpectedly, we did not find any difference in terms of prognosis between the CoNS-HAI and Sa-HAI subgroups. This result could be explained by a lack of power due to the small number of patients in these subgroups. Since all *S. aureus* were treated, we cannot draw a conclusion with regard to the effects of untreated *S. aureus* on the prognosis. In addition, our results suggest that untreated methicillin-resistant CoNS did not worsen morbi-mortality. While the pathogenicity of these microorganisms is well known in several surgical specialities, including orthopaedic, cardio-vascular or neurosurgical cases, the need to treat CoNS in HAI remains unknown. In the current study, 28% of the patients with CoNS samples were not efficiently targeted. These observations illustrate the difficult therapeutic decision for the physician facing CoNS cultures in HAIs. Several hypotheses can explain this observation. The CoNS isolated could be considered as a simple contamination; their pathogenicity in this condition might not be important or there could be a synergy between CoNS and other germs that would be suppressed by the treatment of these other germs. Finally, the CoNS could be as pathogenic as *S. aureus* but not identified due to the insufficient number of patients in this subgroup. This uncertainty leads to suggest targeting these microorganisms in the absence of clinical improvement.

Our study has several limitations. First, it is a retrospective study with inclusions extended over 18 years. However, statistical analysis showed that cases were homogeneously distributed between groups over time, and mortality did not change according to the period of inclusion. Second, many comparisons were performed, which might have increased the probability of type I error. Despite a prolonged duration of inclusion time, we have only collected 29 patients with Sa-HAIs, limiting the value of these observations. Because of the extremely low number of monomicrobial infections, we cannot draw a conclusion about the pathogenicity of staphylococci, especially coagulase-negative staphylococci. It is impossible to differentiate peritoneal colonization from authentic infection. The only clinical surrogates of pathogenic role of the microorganisms cultured from the surgical samples are persistent organ dysfunction and signs of sepsis. This issue leads to cautious consideration of these results and the potential need for antibiotic escalation toward staphylococci for documented therapy in the absence of clinical improvement^21,22^. Finally, the pharmacokinetic characteristics of anti-infective agents is another issue to consider. Even if glycopeptide plasma concentrations were monitored on a routine basis, the peritoneal concentrations are impossible to assess, requiring the careful drawing of conclusions regarding the need to target staphylococci.

In summary, the characteristics of HAIs with positive staphylococci peritoneal cultures differ minimally from those without staphylococci, particularly in terms of clinical presentation. Trends toward decreased morbidity criteria were observed in patients with positive staphylococci peritoneal cultures. However, it is not possible, based on such limited signals, to draw conclusions about the absence of the pathogenicity of staphylococci in HAIs or about the lack of need of antibiotic therapy against these microorganisms. In the absence of clinical improvement after adequate source control, antibiotic toward staphylococci should probably be considered if not included in the empirical therapy. Vancomycin has been largely prescribed for enterococci, while at the same time, targeting the peritoneal staphylococci without knowing if they were real pathogens. Evaluating the efficacy of glycopeptides and new anti-Gram-positive molecules, such as linezolid and daptomycin, in the treatment of intra-abdominal infections could be of interest, with the possible restriction of their use against staphylococci.

## Supplementary Information

Supplementary Information.

## Data Availability

The datasets used and analyzed during the current study are available from the corresponding author on reasonable request.
