# Supplementary material for: Epidemiology, clinical relevance and prognosis of staphylococci in hospital-acquired postoperative intra-abdominal infections: an observational study in intensive care unit
Source: Sci Rep. 2021 Mar 15;11:5884. doi: 10.1038/s41598-021-85443-8 (PMC7960962; doi:10.1038/s41598-021-85443-8)
Supplement: Supplementary file 1 — Supplementary Information. [file 41598_2021_85443_MOESM1_ESM.docx]

**Supplementary Data**

Epidemiology, clinical relevance and prognosis of staphylococci in hospital-acquired postoperative intra-abdominal infections: an observational study in intensive care unit

**Authors**

Kévin BOUSSION, Nathalie ZAPPELLA, Nathalie GRALL, Lara RIBEIRO-PARENTI, Grégory PAPIN, Philippe MONTRAVERS

Supplementary material

The temporal distributions of inclusions were not different between the nS-PI and S-PI groups (2004 [2001-2011] and 2005 [2002-2008], respectively, p=0.50). The 90-day mortality rates were not significantly different according to the period of admission, which were 37.5% (n=65) from 1997 to 2004 and 34% (n=72) from 2005 to 2017 (p=0.35).

Supplementary tables

Table S1. Clinical characteristics of the study population with/without double staphylococci cases

Table S2. Clinical characteristics of the sub populations of Sa-HAI with/without double staphylococci cases.

Table S3. Anti-infective therapies and clinical outcomes in the study population with/without double staphylococci cases

Table S4. Anti-infective therapies and clinical outcomes in the sub population with/without double staphylococci cases

Table S1. Clinical characteristics of the study population with/without double staphylococci cases

| Variables | nS-HAI  n = 293 | S-HAI with double staphylococci  n=87 | S-HAI without double staphylococci cases  n = 85 |
| --- | --- | --- | --- |
| **Demographic characteristics and comorbidities** | | | |
| Age (years), median [25^th^-75^th^] | 61 [50-72] | 62 [45-77] | 61 [45-76] |
| Male gender, n (%) | 164 (56) | 42 (48) | 40 (47) |
| Fatal underlying disease | 91 (31) | 31 (36) | 29 (34) |
| Immunosuppression | 101 (34) | 27 (31) | 25 (29) |
| Cancer | 111 (38) | 27 (31) | 25 (29) |
| Diabetes mellitus | 58 (20) | 11 (13) | 11 (13) |
| **Initial surgery** |  |  |  |
| Emergency procedure | 106 (36) | 39 (45) | 39 (46) |
| Upper gastrointestinal tract surgery | 126 (43) | 47 (54) | 47 (55) * |
| Septic or contaminated surgery | 115 (39) | 33 (38) | 33 (38) |
| Antibiotics before reoperation for PI | 181 (62) | 53 (61) | 52 (61) |
| Interval between initial surgery and reoperation (days) | 7 [4-12] | 7.5 [4-10] | 8 [4-11] |
| **Severity criteria at the time of reoperation** | | | |
| SAPS II score | 49 [37-60] | 46 [33-57] | 46 [33-57] |
| SOFA score | 8 [5-10] | 7 [4-9]* | 7 [4-9] * |
| Hemodynamic failure | 197 (67) | 53 (61) | 51 (60) |
| Respiratory failure | 141 (48) | 32 (37) | 31 (36) |
| Kidney failure | 83 (28) | 21 (24) | 21 (25) |
| **Source of postoperative peritonitis and surgical observations** | | | |
| Anastomotic leak | 107 (37) | 26 (30) | 26 (31) |
| Bowel perforation | 92 (31) | 31 (36) | 31 (36) |
| Abscess | 47 (16) | 18 (21) | 17 (20) |
| No demonstrated cause | 44 (15) | 10 (11) | 9 (11) |
| Gastroduodenal source | 52 (18) | 26 (30)* | 26 (31) * |
| Small bowel source | 75 (26) | 18 (21) | 17 (20) |
| Colonic or rectal source | 79 (28) | 21 (24) | 21 (25) |
| Below transverse mesocolon | 209 (72) | 63 (74) | 61 (72) |
| Generalized peritonitis | 76 (26) | 18 (21) | 17 (20) |

* P<0.05 versus nS-HAI cases

Table S2. Clinical characteristics of the sub populations of Sa-HAI with/without double staphylococci cases.

| Variables | CoNS-HAI  n=58 | Sa-HAI  With double staphylococci  n=29 | Sa-HAI  Without double staphylocci cases  n=27 |
| --- | --- | --- | --- |
| **Demographic characteristics and comorbidities** | | | |
| Age (years), median [25^th^-75^th^] | 63 [46-77] | 57 [37-77] | 56 [35-72] |
| Male gender, n (%) | 32 (55) | 10 (34) | 8 (30)* |
| Fatal underlying disease | 25 (43) | 6 (21)* | 4 (15)* |
| Immunosuppression | 19 (33) | 8 (28) | 6 (22) |
| Cancer | 20 (34) | 7 (24) | 5 (19) |
| Diabetes mellitus | 4 (7) | 7 (24)* | 7 (26) * |
| **Initial surgery** |  |  |  |
| Emergency procedure | 30 (52) | 9 (32)* | 9 (33) |
| Upper gastrointestinal tract surgery | 34 (59) | 13 (45) | 13 (48) |
| Septic or contaminated surgery | 25 (43) | 8 (28) | 8 (30) |
| Antibiotics before reoperation for PI | 39 (67) | 11 (38)* | 11 (41)* |
| Interval between initial surgery and reoperation (days) | 7 [5-11] | 8 [3-10] | 8 [3-10] |
| **Severity criteria at the time of reoperation** | | | |
| SAPS II score | 47 [32-58] | 44 [33-56] | 44 [33-56] |
| SOFA score | 7 [4-9] | 7 [4-10] | 7 [4-10] |
| Hemodynamic failure | 36 (62) | 17 (59) | 15 (56) |
| Respiratory failure | 23 (40) | 9 (31) | 8 (30) |
| Kidney failure | 12 (21) | 9 (31) | 9 (33) |
| **Source of postoperative peritonitis and surgical observations** | | | |
| Anastomotic leak | 16 (28) | 10 (34) | 10 (37) |
| Bowel perforation | 19 (33) | 12 (41) | 12 (44) |
| Abscess | 14 (24) | 4 (14) | 3 (11) |
| No demonstrated cause | 7 (12) | 3 (10) | 2 (7) |
| Gastroduodenal source | 16 (28) | 10 (34) | 10 (37) |
| Small bowel source | 12 (21) | 6 (21) | 5 (19) |
| Colonic or rectal source | 12 (21) | 9 (31) | 9 (33) |
| Below transverse mesocolon | 41 (73) | 22 (76) | 20 (74) |
| Generalized peritonitis | 13 (23) | 5 (17) | 4 (15) |

* P<0.05 versus CoNS-HAI cases

Table S3. Anti-infective therapies and clinical outcomes in the study population with/without double staphylococci cases

| Variables | nS-HAI  n = 293 | S-HAI with double staphylococci°  n = 87 | S-HAI without double staphylococci cases  n = 85 |
| --- | --- | --- | --- |
| **Empirical anti-infective therapy** |  |  |  |
| Monotherapy, n (%) | 57 (19) | 16 (18) | 16 (19) |
| Vancomycin | 129 (44) | 40 (46) | 39 (46) |
| Adequate EAT | 211 (72) | 47 (54)* | 46 (54)* |
| **Documented anti-infective therapy** |  |  |  |
| Monotherapy | 109 (37) | 24 (28) | 24 (28) |
| Carbapenem | 61 (21) | 24 (28) | 24 (28) |
| Piperacillin/tazobactam | 98 (33) | 24 (28) | 23 (27) |
| Vancomycin | 68 (23) | 43 (49)* | 41 (48)* |
| Aminoglycosides | 39 (13) | 14 (16) | 13 (15) |
| Duration (days), median [25^th^-75^th^] | 10 [7-14] | 10 [10-14] | 10 [10-14] |
| De-escalation | 175 (60) | 43 (49) | 42 (49) |
| Escalation | 48 (16) | 31 (36)* | 31 (36)* |
| **Clinical outcomes** |  |  |  |
| Persistent sepsis | 164 (56) | 38 (44)* | 37 (44)* |
| Reoperation | 135 (46) | 35 (40)* | 34 (40) |
| Interval between surgery for PI and reoperation (days) | 4 [2-8] | 6 [4-9]* | 6 [4-9]* |
| Surgical complications | 64 (22) | 9 (10)* | 8 (9)* |
| Medical complications | 45 (15) | 14 (16) | 14 (16) |
| Duration of mechanical ventilation (days)° | 8 [3-17] | 9 [4-22] | 7 [3-14] |
| ICU stay (days)° | 15 [9-28] | 14 [7-23] | 15 [8-27] |
| ICU mortality | 95 (32) | 24 (28) | 23 (27) |
| Hospital mortality | 99 (34) | 26 (30) | 25 (29) |
| 90-day mortality | 104 (35) | 33 (38) | 32 (37) |
| 365-day mortality | 113 (39) | 38 (44) | 37 (44) |

* P<0.05 versus nS-HAI cases

Table S4. Anti-infective therapies and clinical outcomes in the sub population with/without double staphylococci cases

| Variables | CNS-HAI  n = 58 | Sa-HAI with double staphylococci cases  n=29 | Sa-HAI without double staphylococci cases  n = 27 |
| --- | --- | --- | --- |
| **Empirical anti-infective therapy** | | | |
| Monotherapy, n (%) | 12 (21) | 4 (14) | 4 (15) |
| Vancomycin | 25 (43) | 15 (52) | 14 (52) |
| Adequate EAT | 28 (48) | 19 (66) | 18 (67) |
| **Documented anti-infective therapy** | | | |
| Monotherapy | 17 (29) | 7 (24) | 7 (26) |
| Carbapenem | 20 (34) | 4 (14) * | 4 (15) |
| Piperacillin/tazobactam | 12 (21) | 12 (41) * | 11 (41) |
| Vancomycin | 31 (53) | 12 (41) | 10 (37) |
| Aminoglycosides | 8 (14) | 6 (21) | 5 (19) |
| Duration (days), median [25^th^-75^th^] | 10 [8-14] | 10 [10-15] | 10 [10-14] |
| De-escalation | 23 (40) | 20 (69) * | 19 (70) * |
| Escalation | 26 (45) | 5 (17) * | 5 (19) * |
| **Clinical outcomes** | | | |
| Persistent sepsis | 27 (47) | 11 (38) | 10 (37) |
| Reoperation | 23 (40) | 12 (41) | 11 (41) |
| Interval between surgery for PI and reoperation (days) | 5 [3-8] | 7 [5-11] | 7 [4-11] |
| Surgical complications | 5 (9) | 4 (14) | 3 (11) |
| Medical complications | 9 (16) | 5 (17) | 5 (19) |
| Duration of mechanical ventilation (days)° | 7 [4-17] | 5 [2-11] | 6 [2-12] |
| ICU stay (days)° | 15 [8-27] | 14 [7-24] | 15 [7-27] |
| ICU mortality | 18 (33) | 6 (21) | 5 (19) |
| Hospital mortality | 19 (34) | 7 (24) | 6 (22) |
| 90-day mortality | 25 (43) | 8 (28) | 7 (26) |
| 365-day mortality | 28 (48) | 10 (34) | 9 (33) |

* P<0.05 versus CoNS-HAI cases
